# Identification, Prevention and Treatment of Iron Deficiency during the First 1000 Days

**DOI:** 10.3390/nu6104093

**Published:** 2014-10-10

**Authors:** Rachel M. Burke, Juan S. Leon, Parminder S. Suchdev

**Affiliations:** 1 Department of Epidemiology, Rollins School of Public Health, Emory University, 1518 Clifton Rd. NE, Atlanta, GA 30322, USA; 2 Hubert Department of Global Health, Rollins School of Public Health, Emory University, 1518 Clifton Rd. NE, Atlanta, GA 30322, USA; E-Mail: juan.leon@emory.edu; 3 Department of Pediatrics, Emory School of Medicine, 1760 Haygood Dr., Atlanta, GA 30322, USA; E-Mail: psuchde@emory.edu

**Keywords:** iron deficiency, iron supplementation, pediatrics, infants, first 1000 days

## Abstract

Iron deficiency is a global problem across the life course, but infants and their mothers are especially vulnerable to both the development and the consequences of iron deficiency. Maternal iron deficiency during pregnancy can predispose offspring to the development of iron deficiency during infancy, with potentially lifelong sequelae. This review explores iron status throughout these “first 1000 days” from pregnancy through two years of age, covering the role of iron and the epidemiology of iron deficiency, as well as its consequences, identification, interventions and remaining research gaps.

## 1. Introduction

The importance of early life experiences to subsequent health outcomes is increasingly being recognized and studied, with special attention often paid to nutrition and growth. Early nutritional insults can lead to irreversible linear growth restriction (stunting), particularly in the first two years of life [1]. Together with pregnancy, this critical period of “1000 days” is associated with adverse effects much later in the life course, such as increased risk of non-communicable disease, as well as reduced cognitive capacity and economic productivity [2,3]. Given that iron deficiency in the first two years may also cause irreversible deficits in cognitive development, among other potential adverse effects [4,5], we propose that the “first 1000 days” framework can also be useful for the discussion of the identification, prevention and treatment of iron deficiency. Similar to the observation that maternal stunting may lead to intrauterine growth restriction of the fetus, progressing to a stunted infant [6], maternal iron deficiencies may also lead to low iron status for newborns, progressing to iron deficiency in infants [5,7]. This intergenerational cycle provides several potential points for intervention for at-risk mothers and infants. The present review will thus focus on iron deficiency in pregnant, lactating and infant populations (though the first two years of age), describing the role of iron, the epidemiology of iron deficiency, its consequences, identification, interventions and remaining research gaps.

## 2. Iron Metabolism and Requirements 

### 2.1. Iron Metabolism

**Figure 1:**
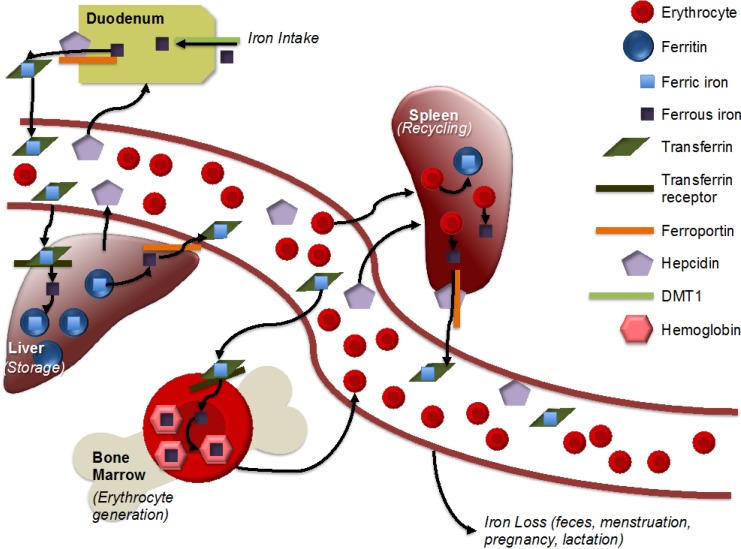
Simplified representation of iron metabolism. Adapted from [12]. Iron is absorbed in the intestine, with non-heme iron being imported by divalent metal transporter 1 (DMT1). Ferrous iron is reduced to ferric iron and then exported by ferroportin. Within an aqueous solution, iron is stored within transferrin. Transferrin-bound iron is imported with the help of the transferrin receptor into the liver, heart and other storage areas, where it is stored within ferritin. Hepcidin, produced by the liver, helps to regulate iron metabolism by binding to ferroportin and, thus, inhibiting iron export. Within the bone marrow, iron is incorporated into hemoglobin for incorporation into erythrocytes. Macrophages recycle iron from erythrocytes, largely in the spleen. There is no mechanism for iron excretion by the kidneys or liver, though small amounts are lost via feces. Menstruation, pregnancy and lactation result in iron loss in women.

Iron is one of the most important micronutrients for human populations, given its central role in key biological processes. One key process is that of tissue oxygenation, which is accomplished by red blood cells (RBCs); generation of RBCs requires hemoglobin, of which iron is a key component [8]. New RBCs are also created to replace RBCs that are lost from normal turnover, shedding (of skin cells or from the intestinal lining) or via hemorrhage [8]. Situations that require an increase in RBCs (such as the increased tissue mass of a growing fetus or infant) will consequently increase iron requirements. Absorption of iron is primarily regulated within the intestine, and once iron has been absorbed, there is no mechanism of excretion from the kidneys or liver [9]. For this reason, iron homeostasis is tightly regulated. After absorption, iron is either stored within ferritin, which keeps the iron in a nonreactive state within cells, or within transferrin, which also keeps the iron in a nonreactive state, but maintains it in aqueous circulation, so that it can be delivered to cells [9]. Within cells, stored iron (as ferritin) can be located in the cytoplasm, nucleus or mitochondria [10]. Iron is transported across cell membranes with the assistance of divalent metal transporter 1 (DMT1; importing function) and ferroportin (exporting function) [9]. Ferroportin can be bound by the protein hepcidin, preventing ferroportin’s export function and, thus, decreasing levels of serum iron (Figure 1) [9]. Whether in serum or within cells, most stored iron is present as ferritin [11].

### 2.2. Requirements in Pregnant and Lactating Women

Iron is especially critical during pregnancy given the rapid cell and tissue development involved in fetal growth. Pregnancy has a net iron cost in the range of 600–800 mg [13,14]. Nearly 300 mg of iron are needed just for the fetus, at least 25 mg for the placenta and nearly 500 mg for the increased volume of red blood cells [15,16]. These ~800 mg of pregnancy-associated iron are a requirement over and above the 230 mg of iron that the woman would have required even if she had not been pregnant, and the 150 mg that she may lose through blood loss at delivery [15,16]. Though this iron expenditure is offset by the lack of menstrual blood loss during this time, the net cost is still high enough that iron recommendations during pregnancy (27 mg per day) far exceed those for non-pregnant, non-lactating women (18 mg per day) [13]. Further, because fetal iron requirements take precedence over maternal needs and storage [14], adequate iron intake is important to both mother and fetus.

While iron recommendations for lactating women are much lower than those for non-pregnant, non-lactating women (9 mg *vs.* 18 mg), this number is based on the assumption of lactation-induced amenorrhea and does not take into account that many women enter or conclude pregnancy with iron insufficiency or deficiency [13].

### 2.3. Requirements in Infants of Zero to 24 Months of Age

Iron requirements for infants under the age of six months are generally not well defined, because needs are difficult to estimate in the context of exclusive breastfeeding [17]. Furthermore, during the first four to six months of age, most infants benefit from iron stores present at birth, most of which are accumulated during the last 10 weeks of gestation [5,18]. Though breastfed infants do not receive large quantities of iron through breast milk (iron concentration < 1 mg/L [19,20,21,22], equivalent to < 0.5 mg per day for a typical infant [23], they are able to absorb a large proportion of this iron [24,25]. For this reason, iron supplementation is typically not recommended for breastfed term infants below the age of six months [23]. However, because preterm infants and low birth weight infants are born with lower birth iron stores, supplementation in the range of 2–4 mg/day is advised in order to avoid development of iron deficiency [4,23]. Supplementation may also be advised for infants that have lower birth iron stores of other etiology. Infant formula typically contains 10–12 mg/L of iron, though this iron is much less bioavailable [26], and evidence to support the benefits of this level of iron intake is inconclusive [23].

For older infants (over six months of age), different methods lead to different estimates of daily requirements, with a commonly used factorial approach generating a recommendation of 11 mg/day for 7–12-month-olds and 7 mg/day for 1–3-year-olds [23]. It is worth noting that requirements likely do not suddenly increase from ~2 mg/day (recommendations for infants <6 months) to ~11 mg/day (recommendations infants 7–12 months), exactly at six months, in a stepwise fashion. This “jump” is rather an artifact generated by the use of the factorial method, which calculates recommendations based upon assumptions about the amount of iron required to accommodate growth (e.g., increased blood volume and tissue mass) and the amount of iron lost (e.g., from skin cells, from intestinal and urinary tracts). However, these assumptions are based on what is known about adult iron metabolism, as no data specific to infant populations is available [17]. Nonetheless, iron requirements certainly do increase from birth through childhood, in order to keep pace with rapid growth during this time, and other methods of calculation (e.g., linear interpolation from breast milk content to adult requirements, extrapolation based on energy expenditure) tend to generate similar results for iron requirements of older infants and toddlers [17].

## 3. Epidemiology of Iron Deficiency

### 3.1. Pregnant and Lactating Women

Iron deficiency (ID) is the most common micronutrient deficiency among pregnant women, leading to iron deficiency anemia (IDA) if uncorrected. However, ID can be difficult to measure in populations due to the lack of availability of field-friendly iron biomarkers (Table 1). In contrast, anemia is less time- and resource-intensive to assess, and thus, anemia is often used as an ersatz surrogate for iron status. However, anemia is neither a sensitive nor a specific indicator of iron status: a loss of up to 20%–30% of body iron would be necessary for some individuals to exhibit anemia based on hemoglobin cut-offs [27], and only up to half of anemia can be attributed to iron deficiency in most settings [28]. However, the prevalence of iron deficiency can be estimated at 2.5-times the prevalence of iron deficiency anemia in many settings [29]. Anemia may affect up to 56% of pregnant women in developing countries, implying a relatively high prevalence of IDA (up to ~25%) and, thus, an even higher prevalence of ID [30]. In settings with endemic malaria, such as many countries in Sub-Saharan Africa, the prevalence of anemia can be much higher—up to 65% in pregnant women and 75% in non-pregnant women of reproductive age [31]. In India, another area of high malaria endemicity, anemia may affect >75% of pregnant women [32], with a prevalence >50%, even in areas with iron supplementation programs [33]. Pregnant women in developed countries are also subject to ID and IDA, with approximately 16% suffering from anemia and more likely suffering from some degree of iron deficiency [29,30]. This is equivalent to approximately 56 million women worldwide [31]. To prevent iron deficiency, it is generally recommended that women enter pregnancy with iron stores of at least 500 mg [5]; however, over 20% of women in developed countries enter pregnancy with extremely low iron stores, with figures in developing countries likely even higher [15].

Although lactating women are not typically considered separately from non-pregnant and non-lactating women in iron deficiency and anemia calculations, the prevalence of anemia among women of reproductive age may provide a useful proxy. Anemia affects 46% of this population in developing countries and 14% in developed countries, indicative of a high prevalence of iron deficiency [30]. Anemia in recently postpartum women may reach 15%–50% in developed country settings and 50%–80% in developing country settings [34].

### 3.2. Infants of Zero to 24 Months of Age

Given that a majority of young term infants are largely protected from iron deficiency by their birth iron stores, iron deficiency is not often considered in infants less than six months of age. However, even in the context of the exclusively breastfed, term infant, iron deficiency and iron deficiency anemia may be observed, with population estimates in the range of 0%–15% (ID) and 0%–4% (IDA) of six-month-old infants in different settings worldwide [35].

Older infants (6–12 and 13–24 months of age) are typically not considered as separate populations in global estimates of ID and IDA. Nonetheless, research indicates that these groups are at even higher risk of ID and IDA than younger infants. In a study of Icelandic infants, Thorisdottir *et al.* found 9% of infants to be iron deficient at 12 months; ID was highest (21%) among infants fed a diet of primarily cow milk, and much lower among infants fed primarily breast milk (2.6%) or formula (1.4%) [36]. In a German study comparing the iron status of infants that had been primarily breastfed during their early months to infants that had been primarily formula fed, 19% of the breastfed infants were iron deficient at seven months, with 4% diagnosed with iron deficiency anemia. At 10 months, 21% suffered ID and 2% suffered IDA [37]. None of the infants who had been formula-fed developed iron deficiencies. The 2006 National Health and Nutrition Survey in Mexico found low iron stores in 32% of children 12–24 months of age and tissue iron deficiency in 19% of the same group [38]. Meanwhile, a population-based study of Indonesian children found that 54% of urban children 6–24 months and 57% of their rural counterparts suffered from anemia, implying a high prevalence of iron deficiency [39]. Data from India indicates that 70%–90% of children 6–59 months of age may be anemic, with prevalence even higher in those under two years of age [40]. Recent demographic and health survey (DHS) data in other developing countries indicate even higher prevalence of anemia in these age groups, with especially high prevalence in West Africa, an area of high malaria endemicity: in Côte d’Ivoire, anemia was found in 90% of 6–9-month-olds, 94% of 9–10-month-olds and 84% of 11–23-month-olds; in Guinea, the prevalence of anemia was about 85% in each of these groups. Similar results were seen in other areas of Sub-Saharan Africa, as well [41].

## 4. Risk Factors for Iron Deficiency

### 4.1. Pregnant and Lactating Women

Pregnant women are especially vulnerable to iron deficiency, not only because of the large quantities of iron required for fetal and placental growth (825 mg for fetus, placenta and increased blood volume [15,16]), but also due to the fact that so many enter pregnancy without adequate iron stores, especially in developing countries [42]. In some of these countries, e.g., India, vegetarianism may be more common due to religious beliefs; these women are also more vulnerable to the development of iron deficiency, because iron is much more efficiently absorbed in heme form (found in animal products). Iron status can also be affected by the intake of other nutrients that may inhibit (e.g., calcium, phytates) or promote (e.g., vitamin C) iron absorption [43]. Other proximal risk factors for low iron status during pregnancy include low intake of bioavailable iron, infections (e.g., intestinal helminthic infections, malaria), multiple pregnancies and adolescent pregnancy, while intermediary factors include low socioeconomic status and membership in certain ethnic groups, depending on the country of residence [5,15,42]. Social and psychological factors may also affect development of iron deficiency anemia, via reducing iron supplementation adherence and compliance [44]. Distal factors, such as food security and access to healthcare, play important roles, as well; “underlying determinants,” such as the existence of anemia control programs (e.g., universal supplementation), fortification policies and the economic situation and agricultural productivity of the locale should also be considered [45]. Iron deficiency factors for lactating women include all of the risk factors identified for pregnant women, in addition to low iron status prior to and during pregnancy, as well as delivery induced bleeding [34]. Obesity has also been associated with iron deficiency in adult populations, with dietary deficiency, elevated blood volume and subclinical inflammation as the suggested mechanisms [46].

### 4.2. Infants of Zero to 24 Months of Age

Given the potentially severe and irreversible consequences of severe iron deficiency and iron deficiency anemia in infants, many studies have sought to assess potential risk factors in addition to treatment and prevention strategies. Infant iron status may be affected by factors arising either prior to the time of birth or following birth, as described below.

#### 4.2.1. Pre- and Peri-Natal Risk Factors

In the absence of supplementation and complementary feeding, young infants are dependent upon iron from only two sources: their birth stores and breast milk or formula. Although breast milk has highly bioavailable iron [24,47], its iron content is not high [19,20,21,22], making birth iron stores critical in the prevention of early iron deficiency [35]. While the importance of birth iron stores is well recognized, determinants of infant birth iron stores remain incompletely understood [48], and the regulation of iron transport from mother to fetus is complex [5,14].

Nonetheless, given the fact that maternal iron status is a known intervention target, multiple studies have assessed the effect of maternal iron status on infant levels, and numerous studies have assessed other outcomes (e.g., low birth weight). There is some suggestion that maternal iron status may affect birth stores in the infant, and infants born to anemic mothers have been shown to be more vulnerable to anemia during their first year of age [49]; correlations between maternal and infant measures of iron status near the time of birth have also been demonstrated [50]. Further, a study of iron status in U.S. infants from birth through 12 months demonstrated that infants maintained their iron ranking over time, again supporting the hypothesis that iron status is influenced by factors that act *in utero* [48]. This is in contrast to some earlier literature, such as a 1961 study by Lanzkowsky *et al.*, which did not demonstrate an effect of maternal anemia on infant anemia [51].

Congruent with the importance of the birth iron stores and the fact that these are generated primarily during the last 10 weeks of gestation, preterm birth and gestational age have consistently been implicated as risk factors for impaired iron status in young infants [52,53,54,55]. Similarly, low birth weight has also been associated with increased risk of iron deficiency in young infants [36,56,57,58].

#### 4.2.2. Postnatal Risk Factors

While pre- and peri-natal risk factors (e.g., low birth weight) can have large effects on infant susceptibility to iron deficiency, infant experiences after birth can also play a large role.

Although iron deficiency is observed in normal-weight, term, exclusively breastfed infants, it is much less common in that population than among preterm or low-birth-weight infants. Furthermore, though breast milk is known to vary widely in exact nutritional content [59], it has generally been assumed that, except in cases of severe maternal nutritional deficiency, breast milk is adequate to meet infant micronutrient requirements, including iron, provided that infants are born with sufficient birth iron stores. Therefore, few studies have studied the impact of postpartum maternal nutritional status on infant nutritional status during the first six months of life, especially in normal birth weight, term infants. Findings of the several studies that have been done appear to be mixed. In a longitudinal study of Peruvian mother-infant pairs, Finkelstein *et al.* found that maternal postpartum hemoglobin and iron status were significantly associated with infant hemoglobin and iron status at two and five months of age, regardless of maternal prenatal supplementation [25]. In contrast, a recent study by Ziegler *et al.* in U.S. infants found no association between maternal and infant plasma ferritin levels at one month postpartum [48], and a randomized clinical trial (RCT) in Turkish mother-infant pairs also found no significant effect of postpartum maternal supplementation on infant parameters of iron status at four months, with the exception of serum iron binding capacity [60].

Once infants reach the age of 4–6 months, iron needs start to outpace iron intake, and stores begin to be exhausted, making infants much more vulnerable to iron deficiency [48]. Infants that grow rapidly, as in the case of low birth weight infants that experience catch-up growth, are also at elevated risk of iron deficiency given their enhanced needs [61]. Further, common complementary foods, especially in developing countries, may be low in iron [35]. Additional factors associated with lower iron intake or absorption (and thus increased risk of iron deficiency) include lower socioeconomic status, cow milk intake and exclusive breastfeeding without additional iron supplementation [36,37,54,58,62,63,64,65,66]. Frequent enteric infections (bacterial, viral or parasitic) are also associated with the development of environmental enteropathy, an intestinal pathology that can negatively affect the absorption of nutrients, such as iron, and cause subsequent deficiencies [67]. Social factors that have been associated with anemia prevalence in young children include rural location [41,68], family structure [69] and sanitation (also related to enteric infections) [45], among others.

## 5. Consequences of Iron Deficiency

### 5.1. Pregnant and Lactating Women

Iron deficiency during pregnancy can have severe consequences, not only for the mother, but also for her infant. Low iron stores and low intake during pregnancy not only cause anemia, associated with weakness, fatigue, reduced cognitive performance and diminished immune response, but may also increase the risk of delivery complications and perinatal maternal mortality [42]. Maternal iron deficiency has also been implicated as a risk factor for preterm delivery, small-for-gestational-age and neonatal mortality [42]. Underscoring the importance of iron to fetal brain development, maternal iron deficiency has also been associated with cognitive and behavioral deficits [42,70,71], likely mediated through reduced birth iron stores and subsequent iron deficiency in the infant [5]. Iron deficiency during pregnancy also increases the risk of iron deficiency anemia during lactation [13]. Among lactating women, iron deficiency has the same effects as on non-pregnant, non-lactating women of reproductive age: increased risk of iron deficiency anemia, reduced work and mental capacity, increased risk of postpartum depression and other emotional disorders, as well as reduced quality of mother-child interactions [42].

### 5.2. Infants Zero to 24 Months

Infants are vulnerable to the effects of low iron status, even before the first moments of birth: low birth iron stores have been associated with both iron deficiency and with increased risk of cognitive and psychomotor developmental deficits later in infancy [35]. Iron deficiency that develops later in infancy and leads to iron deficiency anemia has similarly been associated with impaired cognitive, behavioral and motor development; the effect of iron deficiency without iron deficiency anemia is less clear [61,66]. Iron deficiency in young children has also been associated with elevated blood lead levels, with some evidence of a causative relationship, wherein iron deficiency makes children more vulnerable to higher blood lead levels; elevated blood lead levels, like iron deficiency, can also cause cognitive impairment [72]. The cognitive deficits of iron deficiency may be irreversible, even if iron supplementation is begun within the critical period of zero to 24 months [4,7].

## 6. Screening and Measurement of Iron Deficiency

### 6.1. Commonly Used Indicators

Several biomarkers are used to assess iron deficiency, and each has its own advantages and disadvantages (Table 1). While hemoglobin is commonly used to assess anemia, it is neither a sensitive nor a specific indicator of iron status. For instance, hemoglobin (Hb) levels may not fall below anemia cut-offs until iron stores fall by up to one-third, and anemia can also be caused by several other micronutrient deficiencies (e.g., B12, folate) or conditions [11,27,73]. Serum ferritin (SF), a marker of iron storage, has the advantage of being a sensitive indicator of iron deficiency, but because it is increased in the presence of inflammation, ferritin is not a specific indicator of iron deficiency [11,27,73]. Transferrin saturation (Tfs), a marker of circulating iron, has also been widely used, but levels are depressed in the presence of inflammation, decreasing its specificity [74,75]. Though less affected by inflammation than SF or Tfs, soluble transferrin receptor (sTfR) levels begin to change relatively late in iron deficiency; further, levels can also be affected by other causes of altered rates of red blood cell generation [11,27,73]. Nonetheless, this marker may be preferred for infant populations with high levels of background infection, as it has been shown to have good accuracy in this setting [76]. Zinc protoporphyrin (ZPP) is another indicator and can reflect a shortage of iron in the last step prior to hemoglobin formation; however, it is not a specific indicator of iron status [11,27,73]. The ratio of TfR to SF can be a useful marker of iron status, but is again limited by SF’s response to inflammation and has also not been fully validated in children or infants [27]. Hepcidin is a liver-produced hormone that is active in iron homeostasis and, thus, has potential as a biomarker of iron status and function [5]. Levels of hepcidin are decreased in conditions leading to or resulting from iron deficiency (e.g., erythropoiesis), and increased in conditions of iron sufficiency (high iron stores) or inflammation [5,75]. However, the relationship of hepcidin to inflammation, as well as the fact that normative levels are still ill-defined, limits the utility of hepcidin alone in the definition of iron status [5]. Reticulocyte hemoglobin (CHr; mean cellular hemoglobin content of reticulocytes) has also recently been recommended for the diagnosis of iron deficiency in infant populations [23]. CHr is a measure of the iron incorporated into the hemoglobin of red blood cells and, thus, provides a fairly direct measure of iron availability to cells; it has also been shown to be highly accurate when compared with other biomarkers [77]. While this marker also has the advantage of not being affected by inflammation, the required assay is not yet widely available [23]. Thus, it is most useful to assess iron status with several markers, as opposed to just one. For instance, in CDC analysis of the National Health and Nutrition Examination Survey (NHANES), iron deficiency is usually defined as abnormal values on at least two of the following three indicators: ZPP, SF, sTfR [78].

**Table 1:** Summary of iron indicators. Iron indicators are ranked from easiest and most economical to measure, to most expensive and most invasive.

Biomarker	Advantages	Limitations	Normal Range/Cut-offs
Hemoglobin (Hb)	Easy, economical to measure (can be assessed with handheld device) Good screening tool for severe iron deficiency	Neither sensitive nor specific for iron status Better measure of function rather than status	Pregnant women: anemia <11.0 g/dL (1T, 3T) or <10.5 g/dL (2T)* Newborns: anemia <13.0 g/dL (venous), <14.5 g/dL (capillary) Infants 6–24 months: anemia <11.0 g/dL
Hematocrit (Hct)	Relatively easy to measure	Provides no additional information above Hb	Pregnant women: anemia <33% Infants 6–24 months: anemia <32%
Red blood cell indices (mean cell volume (MCV), red cell distribution width (RDW))	Low MCV and increased RDW characteristic of iron deficient erythropoiesis Useful clinically	Late finding, not representative of iron status	MCV ◦ Pregnant and lactating women: <82 fl (Femtoliters) ◦ Infant reference ranges (age-dependent): ▪ Neonates: 100–112 fl ▪ <2 months: 85–98 fl ▪ 2–12 months: 73–84 fl ▪ 12–24 months: 72–85 fl RDW ◦ Abnormal: <11.5%, >14.5%
Serum or plasma iron	Measure of circulating iron	Easily contaminated by iron from other sources Variation by time of day, post-prandial state Does not detect iron in Hb	Adults: <40–50 µg/dL Infants <24 months: <50–60 µg/dL
Serum ferritin (SF)	Sensitive indicator of iron deficiency ◦ Proportional to liver stores of iron Responds well to iron interventions	Increases with the acute phase response (not specific in the presence of inflammation)	Pregnant women: <12.0 µg/L (1T) Reference range (women): 0–230 µg/L (trimester-dependent) Newborns: <34.0 µg/L (cord blood) Infants 6–24 months: <12.0 µg/L
Transferrin saturation (Tfs)	Marker of circulating iron	Levels are depressed by inflammation	Pregnant women: <16% Infants <24 months: <10%
Transferrin receptor (TfR)	Less sensitive to inflammation than SF ◦ Useful in populations with high levels of background infection	Not very sensitive; levels change only late in ID Not as specific as other measures; other conditions may cause restriction of iron to RBCs	Pregnant women: >8.5 mg/L or >4.4 mg/L Infants <24 months: >20 mg/L
TfR:SF ratio	Proportional to stored iron or iron deficit Sensitive indicator of response to iron supplementation	Vulnerable to effects of inflammation on SF Not validated in children or infants Assay dependent (based on Ramco assay for TfR)	Pregnant women: >500 consistent with iron deficiency or depleted iron stores Can be used to calculate body iron stores: -[log (TfR/ferritin ratio) −2.8229]/0.1207 ◦ Negative values defined as tissue iron deficit
Total iron binding capacity (TIBC)	More stable than other measures Measures iron-binding sites on transferrin	Changes only with depletion of iron stores Not typically used in newborns	Adults: >400 µmg/dL
Zinc protoporphyrin (ZPP)	Sensitive indicator of severe iron deficiency, but not of moderate iron deficiency Can be measured with very little blood volume	Not specific as levels can be increased due to lead poisoning, inflammation, and other situations Cut-off levels not well established for infant populations	Pregnant women: >70 µg/dL RBCs (1T) Infants <24 months: >70–80 µg/dL RBCs
Hepcidin (Hep)	Reflects iron homeostasis May be measured in blood or in urine	Also increases in conditions of inflammation Normative levels not well defined	Pregnant and lactating women: Mean levels immediately prior and following delivery have ranged 2.5–17.5 µg/dL Newborns: Mean levels in cord blood have ranged 48.5–69.3 µg/dL
Reticulocyte hemoglobin (CHr)	Measure of iron availability to cells Not affected by inflammation	Assay not yet widely available	Adults: reference range 28–35 pg/L Infants <24 months: reference range 23–35 pg/L
Stainable bone marrow	Gold standard for diagnosis of iron deficiency	Invasive Subject to observer error	Units: Observer assesses stained iron content according to a semi-quantitative scale

* 1T: first trimester; 2T: second trimester; 3T: third trimester. References: [5,11,23,27,28,79,80,81,82,83,84,85,86,87,88].

### 6.2. Impact of Inflammation on Indicators of Iron Status

Measurement of iron status is further complicated by the fact that infection and its resulting inflammation, even at a sub-clinical level, can affect serum levels of markers commonly used to assess iron deficiency [75]. Though this effect plays a role in the assessment of most micronutrients, it is particularly important for the measurement of iron status. While serum iron and transferrin (which binds iron and becomes low in the case of iron deficiency) are depressed in the presence of inflammation, ferritin (which decreases with iron deficiency) rises in the presence of inflammation [75]. These changes are thought to reflect an evolutionary effort to sequester iron away from pathogen use during infection (for instance, by bacterial or parasitic agents) [89]. Other measures of iron status are also affected by infection and inflammation.

The body’s initial inflammatory reaction (the acute phase response; APR) activates a number of proteins (acute phase proteins; APP) and other mediators that help the body to recover from the instigating trauma [75]. This response typically lasts nine to 10 days, with the levels of different APP varying over this time. C-reactive protein (CRP) is one of the first to peak, reaching its maximum within one to two days after the initial insult, and with a half-life of two days. Alpha-1-glycoprotein (AGP) is also commonly measured, but is slower to peak than CRP (maximum reached at four to five days) and has a longer half-life, as well (5.2 days). While ferritin is itself considered an APP, the exact biological mechanisms of the APR-driven changes in other iron biomarkers are not as well understood. However, given that these effects have been widely observed, it is critical to adjust for inflammation status when assessing prevalence of iron deficiency in a population [90].

While many nutritional studies commonly use C-reactive protein (CRP) to adjust for inflammation (e.g., by calculating correction factors based on nutrient levels in inflamed *vs*. non-inflamed groups), this may not be sufficient to fully correct for the effect of inflammation on iron status, given the short half-life of CRP and the timing of its peak relative to inflammation-induced changes in iron measures. Transferrin and serum iron both fall relatively rapidly in the presence of inflammation, while ferritin rises quickly, much like CRP. However, other measures of iron status (and anemia status), such as hematocrit, hemoglobin and zinc protoporphyrin (ZPP), change much more slowly, and even ferritin takes longer to return to normal levels than CRP does [75]. Thus, adjustment for inflammation based solely on CRP levels may not fully account for the changes in micronutrient levels that occur as a result of the APR. More recently, investigators have measured both CRP and AGP in order to categorize the stage of inflammation with more precision, thus adjusting for four different inflammation groups instead of a simple dichotomy [75,91]. While other proteins, such as alpha-1-antichymotrypsin (ACT) and ceruloplasmin, have also been measured and used in some studies to adjust micronutrient status for inflammation [92,93], they are not as commonly used as either AGP or CRP.

## 7. Interventions

### 7.1. Pregnant and Lactating Women

The most common and effective intervention to combat iron deficiency in pregnant women is supplementation with iron, often combined with folic acid (given to prevent neural tube defects). As described in previous sections, given the number of women that enter pregnancy with insufficient iron stores, iron supplementation may need to begin prior to conception to ensure prevention of maternal iron deficiency. Indeed, the World Health Organization (WHO) recommends weekly iron-folic acid supplementation programs in areas with a high prevalence of anemia in women of reproductive age (WRA) [94]. Daily iron supplementation is generally recommended for pregnant women worldwide, though in some developed country settings, universal iron supplementation is not recommended due to uncertain benefit and adverse effects (e.g., GI upset) in iron-replete women [5,95]. The success of iron supplementation programs is also often limited by poor adherence (as low as 50% in some settings [96]), which can be associated with gastrointestinal side effects, as well as lack of reliable access to supplements [97,98]. Further, these supplements often incorporate iron of lower bioavailability [98]. While fortification of foods would also help reduce iron deficiency among pregnant women, programs are sparsely implemented in many countries and may be insufficient to meet iron needs without additional supplementation [99]. Multiple micronutrient powders containing iron may also have good potential to prevent iron deficiencies, but more research is needed in order to determine their level of benefit [100,101]. The risk of postpartum iron deficiency can also be reduced by antenatal iron supplementation, as well as by prevention of perinatal hemorrhage [102].

### 7.2. Infants Zero–24 Months of Age

Infants, particularly preterm or low-birth-weight infants, will also benefit from iron supplementation, regularly recommended for these high-risk populations [4]; infant iron supplementation is often provided in liquid form and has been shown to be effective in reducing anemia [45], as well as iron deficiency [4,54]. However, these drops have several disadvantages: they frequently cause gastrointestinal side effects and also create the potential for overdose [103]. Multiple micronutrient powders (MNPs) containing iron for home fortification of complementary food have also been shown to be highly effective in reducing anemia, as well as iron deficiency in infant populations 6–24 months of age [103]. These have the advantage of providing not only iron, but other critical micronutrients, as well. Further, MNPs are easy for mothers to use and incorporate into complementary feeding and carry a lower risk of overdose, as they are packaged in single-dose packets [104].

Nonetheless, iron supplementation (in various forms) has been associated with certain risks, particularly in iron-replete populations. Initial controversy was sparked in 2006, when an RCT of iron supplementation of preschoolers in malaria-endemic Zanzibar was published showing an increased risk of severe adverse events and death in the supplementation arm [105]. Though a Cochrane review has since concluded that there is no excess risk in settings with regular malaria surveillance and control [106], the development of an appropriate universal policy remains a topic of discussion [107]. Iron supplementation of iron-replete infants has also been associated with reduced linear growth and increased risk of other infections in some populations [61,103,108].

Delayed cord clamping has also been shown to have a protective effect against iron deficiency in infants: a recent Cochrane review found that infants whose cord clamping was not delayed were over twice as likely to later be found iron deficient as compared to infants whose cord clamping was delayed [109]; this is likely related to the amount of blood transferred to the infant during this time, with the delayed cord clamping allowing approximately 30% more blood to be transferred to the infant [5].

## 8. Research Needs

Although our understanding of iron function and metabolism and ability to assess and improve iron status in diverse populations has vastly improved in the last decades, much progress remains to be made. In particular, several gaps remain in the assessment of iron status, especially in resource-limited settings, as well as in the safe and cost-effective prevention and treatment of iron deficiency.

While it is now widely recognized that inflammation affects commonly used markers of iron status, there is still a lack of consensus on how to best adjust for these effects. There are numerous markers of inflammation that could be measured, each with different behavior (signaling a different role or stage in the inflammatory process), and each requiring a different assay, with some assays being more expensive or less widely available than others. Appropriate cut-offs for each inflammatory biomarker are also not well defined. Furthermore, the exact approach to adjust for inflammation using these markers is also still under consideration: while correction factors are most commonly used, other mechanisms of adjustment, such as linear regression, may also be useful [90].

Furthermore, currently available iron biomarkers have numerous other limitations apart from sensitivity to inflammation: they are not field-friendly, must be used in concert (*i.e.*, the use of a single biomarker to assess iron status is not recommended) and do not correlate well to iron function or iron exposure. Accurate measures of iron function are critical in order to be able to better understand the impact of supplementation and the effects of deficiency. For instance, brain imaging has been explored as a potential way to elucidate the mechanisms behind iron’s role in early cognitive development [110]; this and other functional biomarkers should be further researched. Additionally, biomarkers of iron exposure would be useful during pregnancy, since pregnancy often depletes maternal iron stores in the last trimester, when the fetus is building its birth iron store [74]. Lastly, measures of iron status also need to be better defined and validated for specific populations, such as young infants.

In terms of prevention and treatment of iron deficiency, additional research should assess the risk-benefit of iron supplementation and clearly communicate this to policy makers. This is particularly important in the supplementation of young infants, given the mixed evidence as to the potential adverse effects of supplementation on growth and vulnerability to infection. The risk-benefit calculation for supplementation of pregnant and lactating women is less ambiguous, though more work needs to be done on ways to improve adherence and bioavailability of supplements. Further, the potential advantages of moving away from a supplementation-focused approach to a food-based approach (e.g., staple food fortification, promotion of dietary diversity) should be explored. Nutrition-sensitive interventions, e.g., home gardens, biofortification or conditional cash transfers, could also support a whole-foods-based approach, while simultaneously addressing social and economic determinants of iron status discussed above; however, additional research is needed to determine the effectiveness and feasibility of such programs for improvement of iron status [111].

## 9. Conclusions and Recommendations

Iron deficiency is undeniably a critical public health issue given its high prevalence and potentially life-altering consequences. Infants are particularly vulnerable to iron deficiency due to their rapid growth, and consequences of iron deficiency in this population can be wide-ranging and long lasting. However, because of the links between maternal iron status and neonatal iron status, interventions on infants alone will be insufficient to reduce levels of infant iron deficiency; the improvement of maternal iron status (before, during and after pregnancy) is also critical. While many advances have been made, knowledge gaps still remain, not only in the best approach to intervention, but also in the most correct approach to screening and diagnosis of iron deficiency itself.

This review summarizes the importance, development and diagnosis and treatment of iron deficiency in maternal and infant populations. We have placed special emphasis on the influence of early and fetal life experiences on iron deficiency and its consequences, as well as the interplay between mother and infant before and after birth. With our treatment of iron deficiency from fetal life through the first two years of age, we hope that we have demonstrated the “first 1000 days” framework to be highly useful in considering iron status and planning interventions.

## References

[1] Victora C.G., de Onis M., Hallal P.C., Blossner M., Shrimpton R. (2010). Worldwide timing of growth faltering: Revisiting implications for interventions. Pediatrics.

[2] Black R.E., Victora C.G., Walker S.P., Bhutta Z.A., Christian P., de Onis M., Ezzati M., Grantham-McGregor S., Katz J., Martorell R. (2013). Maternal and child undernutrition and overweight in low-income and middle-income countries. Lancet.

[3] Victora C.G., Adair L., Fall C., Hallal P.C., Martorell R., Richter L., Sachdev H.S. (2008). Maternal and child undernutrition: Consequences for adult health and human capital. Lancet.

[4] Berglund S., Domellof M. (2014). Meeting iron needs for infants and children. Curr. Opin. Clin. Nutr. Metab. Care.

[5] Cao C., O’Brien K.O. (2013). Pregnancy and iron homeostasis: An update. Nutr. Rev..

[6] Martorell R., Zongrone A. (2012). Intergenerational influences on child growth and under nutrition. Paediatr. Perinat. Epidemiol..

[7] Radlowski E.C., Johnson R.W. (2013). Perinatal iron deficiency and neurocognitive development. Front. Human Neurosci..

[8] Miller J.L. (2013). Iron deficiency anemia: A common and curable disease. Cold Spring Harbor Perspect. Med..

[9] Andrews N.C. (2008). Forging a field: The golden age of iron biology. Blood.

[10] MacKenzie E.L., Iwasaki K., Tsuji Y. (2008). Intracellular iron transport and storage: From molecular mechanisms to health implications. Antioxid. Redox Signal..

[11] WHO (2004). Assessing the iron status of populations: Including literature reviews. Report of a Joint World Health Organization/Centers for Disease Control and Prevention Technical Consultation on the Assessment of Iron Status at the Population Level.

[12] Escobar-Morreale H.F. (2012). Iron metabolism and the polycystic ovary syndrome. Trends Endocrinol. Metab..

[13] Picciano M.F. (2003). Pregnancy and lactation: Physiological adjustments, nutritional requirements and the role of dietary supplements. J. Nutr..

[14] Gambling L., Lang C., McArdle H.J. (2011). Fetal regulation of iron transport during pregnancy. Am. J. Clin. Nutr..

[15] Bothwell T.H. (2000). Iron requirements in pregnancy and strategies to meet them. Am. J. Clin. Nutr..

[16] Beaton G.H. (2000). Iron needs during pregnancy: Do we need to rethink our targets?. Am. J. Clin. Nutr..

[17] Bender D.A. (2003). Do we really know vitamin and mineral requirements for infants and children?. J. R. Soc. Promot. Health.

[18] Siddappa A.M., Rao R., Long J.D., Widness J.A., Georgieff M.K. (2007). The assessment of newborn iron stores at birth: A review of the literature and standards for ferritin concentrations. Neonatology.

[19] Marin G.H., Mestorino N., Errecalde J., Huber B., Uriarte A., Orchuela J. (2012). Personalised iron supply for prophylaxis and treatment of pregnant women as a way to ensure normal iron levels in their breast milk. J. Med. Life.

[20] Nikniaz L., Mahdavi R., Gargari B.P., Gayem Magami S.J., Nikniaz Z. (2011). Maternal body mass index, dietary intake and socioeconomic status: Differential effects on breast milk zinc, copper and iron content. Health Promot. Perspect..

[21] Silvestre D., Martinez-Costa C., Lagarda M.J., Brines J., Farre R., Clemente G. (2001). Copper, iron, and zinc contents in human milk during the first three months of lactation: A longitudinal study. Biol. Trace Elem. Res..

[22] Yamawaki N., Yamada M., Kan-no T., Kojima T., Kaneko T., Yonekubo A. (2005). Macronutrient, mineral and trace element composition of breast milk from Japanese women. J. Trace Elem. Med. Biol..

[23] Baker R.D., Greer F.R. (2010). Diagnosis and prevention of iron deficiency and iron-deficiency anemia in infants and young children (0–3 years of age). Pediatrics.

[24] Saarinen U.M., Siimes M.A., Dallman P.R. (1977). Iron absorption in infants: High bioavailability of breast milk iron as indicated by the extrinsic tag method of iron absorption and by the concentration of serum ferritin. J. Pediatr..

[25] Finkelstein J.L., O’Brien K.O., Abrams S.A., Zavaleta N. (2013). Infant iron status affects iron absorption in Peruvian breastfed infants at 2 and 5 months of age. Am. J. Clin. Nutr..

[26] Lonnerdal B., Kelleher S.L. (2007). Iron metabolism in infants and children. Food Nutr. Bull..

[27] Zimmermann M.B. (2008). Methods to assess iron and iodine status. Br. J. Nutr..

[28] Cameron B.M., Neufeld L.M. (2011). Estimating the prevalence of iron deficiency in the first two years of life: Technical and measurement issues. Nutr. Rev..

[29] WHO (2001). Iron deficiency anaemia: Assessment, prevention, and control. A Guide for Programme Managers.

[30] McLean E., Cogswell M., Egli I., Wojdyla D., de Benoist B. (2009). Worldwide prevalence of anaemia, WHO vitamin and mineral nutrition information system, 1993–2005. Public Health Nutr..

[31] WHO (2008). Worldwide Prevalence of Anaemia 1993–2005. WHO Global Database on Anaemia.

[32] Qureshi I.A., Arlappa N., Qureshi M.A. (2014). Prevalence of malaria and anemia among pregnant women residing in malaria-endemic forest villages in India. Int. J. Gynaecol. Obstetr..

[33] Menon K.C., Ferguson E.L., Thomson C.D., Gray A.R., Zodpey S., Saraf A., Das P.K., Pandav C.S., Skeaff S.A. (2014). Iron status of pregnant Indian women from an area of active iron supplementation. Nutrition.

[34] Milman N. (2011). Postpartum anemia I: Definition, prevalence, causes, and consequences. Ann. Hematol..

[35] Ziegler E.E., Nelson S.E., Jeter J.M. (2011). Iron supplementation of breastfed infants. Nutr. Rev..

[36] Thorisdottir A.V., Ramel A., Palsson G.I., Tomassson H., Thorsdottir I. (2013). Iron status of one-year-olds and association with breast milk, cow’s milk or formula in late infancy. Eur. J. Nutr..

[37] Dube K., Schwartz J., Mueller M.J., Kalhoff H., Kersting M. (2010). Iron intake and iron status in breastfed infants during the first year of life. Clin. Nutr..

[38] Morales-Ruan Mdel C., Villalpando S., Garcia-Guerra A., Shamah-Levy T., Robledo-Perez R., Avila-Arcos M.A., Rivera J.A. (2012). Iron, zinc, copper and magnesium nutritional status in Mexican children aged 1 to 11 years. Salud Publica de Mexico.

[39] Sandjaja S., Budiman B., Harahap H., Ernawati F., Soekatri M., Widodo Y., Sumedi E., Rustan E., Sofia G., Syarief S.N. (2013). Food consumption and nutritional and biochemical status of 0.5–12-year-old Indonesian children: The SEANUTS study. Br. J. Nutr..

[40] Sachdev H.P., Gera T. (2013). Preventing childhood anemia in India: Iron supplementation and beyond. Eur. J. Clin. Nutr..

[41] Statcompiler: Building Tables with DHS Data. http://www.statcompiler.com/.

[42] Milman N. (2011). Iron in pregnancy: How do we secure an appropriate iron status in the mother and child?. Ann. Nutr. Metab..

[43] Collings R., Harvey L.J., Hooper L., Hurst R., Brown T.J., Ansett J., King M., Fairweather-Tait S.J. (2013). The absorption of iron from whole diets: A systematic review. Am. J. Clin. Nutr..

[44] Tran T.D., Biggs B.A., Tran T., Casey G.J., Hanieh S., Simpson J.A., Dwyer T., Fisher J. (2013). Psychological and social factors associated with late pregnancy iron deficiency anaemia in rural Viet Nam: A population-based prospective study. PLoS One.

[45] Pasricha S.R., Drakesmith H., Black J., Hipgrave D., Biggs B.A. (2013). Control of iron deficiency anemia in low- and middle-income countries. Blood.

[46] Nikonorov A.A., Skalnaya M.G., Tinkov A.A., Skalny A.V. (2014). Mutual interaction between iron homeostasis and obesity pathogenesis. J. Trace Elem. Med. Biol..

[47] Hicks P.D., Zavaleta N., Chen Z., Abrams S.A., Lonnerdal B. (2006). Iron deficiency, but not anemia, upregulates iron absorption in breast-fed Peruvian infants. J. Nutr..

[48] Ziegler E.E., Nelson S.E., Jeter J.M. (2014). Iron stores of breastfed infants during the first year of life. Nutrients.

[49] Scholl T.O. (2011). Maternal iron status: Relation to fetal growth, length of gestation, and iron endowment of the neonate. Nutr. Rev..

[50] Shao J., Lou J., Rao R., Georgieff M.K., Kaciroti N., Felt B.T., Zhao Z.Y., Lozoff B. (2012). Maternal serum ferritin concentration is positively associated with newborn iron stores in women with low ferritin status in late pregnancy. J. Nutr..

[51] Lanzkowsky P. (1961). The influence of maternal iron-deficiency anaemia on the haemoglobin of the infant. Arch. Dis. Child..

[52] Ozdemir H., Akman I., Demirel U., Coskun S., Bilgen H., Ozek E. (2013). Iron deficiency anemia in late-preterm infants. Turk. J. Pediatr..

[53] Hay G., Refsum H., Whitelaw A., Melbye E.L., Haug E., Borch-Iohnsen B. (2007). Predictors of serum ferritin and serum soluble transferrin receptor in newborns and their associations with iron status during the first 2 years of life. Am. J. Clin. Nutr..

[54] Berglund S., Westrup B., Domellof M. (2010). Iron supplements reduce the risk of iron deficiency anemia in marginally low birth weight infants. Pediatrics.

[55] Haga P. (1980). Plasma ferritin concentrations in preterm infants in cord blood and during the early anaemia of prematurity. Acta Paediatr. Scand..

[56] Yang Z., Lonnerdal B., Adu-Afarwuah S., Brown K.H., Chaparro C.M., Cohen R.J., Domellof M., Hernell O., Lartey A., Dewey K.G. (2009). Prevalence and predictors of iron deficiency in fully breastfed infants at 6 mo of age: Comparison of data from 6 studies. Am. J. Clin. Nutr..

[57] Mamiro P.S., Kolsteren P., Roberfroid D., Tatala S., Opsomer A.S., Van Camp J.H. (2005). Feeding practices and factors contributing to wasting, stunting, and iron-deficiency anaemia among 3–23-month old children in Kilosa district, rural Tanzania. J. Health Popul. Nutr..

[58] Monterrosa E.C., Frongillo E.A., Vasquez-Garibay E.M., Romero-Velarde E., Casey L.M., Willows N.D. (2008). Predominant breast-feeding from birth to six months is associated with fewer gastrointestinal infections and increased risk for iron deficiency among infants. J. Nutr..

[59] Powers H.J. (1997). Vitamin requirements for term infants: Considerations for infant formulae. Nutr. Res. Rev..

[60] Baykan A., Yalcin S.S., Yurdakok K. (2006). Does maternal iron supplementation during the lactation period affect iron status of exclusively breast-fed infants?. Turk. J. Pediatr..

[61] Domellof M., Braegger C., Campoy C., Colomb V., Decsi T., Fewtrell M., Hojsak I., Mihatsch W., Molgaard C., Shamir R. (2014). Iron requirements of infants and toddlers. J. Pediatr. Gastroenterol. Nutr..

[62] Van de Lagemaat M., Amesz E.M., Schaafsma A., Lafeber H.N. (2013). Iron deficiency and anemia in iron-fortified formula and human milk-fed preterm infants until 6 months post-term. Eur. J. Nutr..

[63] Maguire J.L., Salehi L., Birken C.S., Carsley S., Mamdani M., Thorpe K.E., Lebovic G., Khovratovich M., Parkin P.C. (2013). Association between total duration of breastfeeding and iron deficiency. Pediatrics.

[64] Kim H.J., Kim D.H., Lee J.E., Kwon Y.S., Jun Y.H., Hong Y.J., Kim S.K. (2013). Is it possible to predict the iron status from an infant's diet history?. Pediatr. Gastroenterol. Hepatol. Nutr..

[65] Iwai Y., Takanashi T., Nakao Y., Mikawa H. (1986). Iron status in low birth weight infants on breast and formula feeding. Eur. J. Pediatr..

[66] Kazal L.A. (2002). Prevention of iron deficiency in infants and toddlers. Am. Family Phys..

[67] Dewey K.G., Mayers D.R. (2011). Early child growth: How do nutrition and infection interact?. Matern. Child Nutr..

[68] Pita G.M., Jimenez S., Basabe B., Garcia R.G., Macias C., Selva L., Hernandez C., Cruz M., Herrera R., O’Farrill R. (2014). Anemia in children under five years old in eastern Cuba, 2005–2011. MEDICC Rev..

[69] Schmeer K.K. (2013). Family structure and child anemia in Mexico. Soc. Sci. Med..

[70] Chang S., Zeng L., Brouwer I.D., Kok F.J., Yan H. (2013). Effect of iron deficiency anemia in pregnancy on child mental development in rural China. Pediatrics.

[71] Tran T.D., Biggs B.A., Tran T., Simpson J.A., Hanieh S., Dwyer T., Fisher J. (2013). Impact on infants’ cognitive development of antenatal exposure to iron deficiency disorder and common mental disorders. PLoS One.

[72] Kordas K. (2010). Iron, lead, and children’s behavior and cognition. Ann. Rev. Nutr..

[73] Iron-Status Indicators. http://www.cdc.gov/nutritionreport/99-02/pdf/nr_ch3.pdf.

[74] Raiten D.J., Namaste S., Brabin B., Combs G., L’Abbe M.R., Wasantwisut E., Darnton-Hill I. (2011). Executive summary—Biomarkers of nutrition for development: Building a consensus. Am. J. Clin. Nutr..

[75] Thurnham D., McCabe G. (2012). Influence of Infection and Inflammation on Biomarkers of Nutritional Status with an Emphasis on Vitamin A and Iron.

[76] Aguilar R., Moraleda C., Quinto L., Renom M., Mussacate L., Macete E., Aguilar J.L., Alonso P.L., Menendez C. (2012). Challenges in the diagnosis of iron deficiency in children exposed to high prevalence of infections. PLoS One.

[77] Brugnara C., Schiller B., Moran J. (2006). Reticulocyte hemoglobin equivalent (ret he) and assessment of iron-deficient states. Clin. Lab. Haematol..

[78] Looker A.C., Dallman P.R., Carroll M.D., Gunter E.W., Johnson C.L. (1997). Prevalence of iron deficiency in the United States. J. Am. Med. Assoc..

[79] Galesloot T.E., Vermeulen S.H., Geurts-Moespot A.J., Klaver S.M., Kroot J.J., van Tienoven D., Wetzels J.F., Kiemeney L.A., Sweep F.C., den Heijer M. (2011). Serum hepcidin: Reference ranges and biochemical correlates in the general population. Blood.

[80] Gyarmati B., Szabo E., Szalay B., Czuczy N., Toldi G., Cseh A., Vasarhelyi B., Takats Z. (2011). Serum maternal hepcidin levels 3 days after delivery are higher compared to those measured at parturition. J. Obstetr. Gynaecol. Res..

[81] Muller K.F., Lorenz L., Poets C.F., Westerman M., Franz A.R. (2012). Hepcidin concentrations in serum and urine correlate with iron homeostasis in preterm infants. J. Pediatr..

[82] Rehu M., Punnonen K., Ostland V., Heinonen S., Westerman M., Pulkki K., Sankilampi U. (2010). Maternal serum hepcidin is low at term and independent of cord blood iron status. Eur. J. Haematol..

[83] Schulze K.J., Christian P., Ruczinski I., Ray A.L., Nath A., Wu L.S., Semba R.D. (2008). Hepcidin and iron status among pregnant women in Bangladesh. Asia Pac. J. Clin. Nutr..

[84] Young M.F., Griffin I., Pressman E., McIntyre A.W., Cooper E., McNanley T., Harris Z.L., Westerman M., O'Brien K.O. (2012). Maternal hepcidin is associated with placental transfer of iron derived from dietary heme and nonheme sources. J. Nutr..

[85] Simavli S., Derbent A.U., Uysal S., Turhan N.O. (2014). Hepcidin, iron status, and inflammation variables among healthy pregnant women in the Turkish population. J. Mater. Fetal Neonatal Med..

[86] Ronnenberg A.G., Wood R.J., Wang X., Xing H., Chen C., Chen D., Guang W., Huang A., Wang L., Xu X. (2004). Preconception hemoglobin and ferritin concentrations are associated with pregnancy outcome in a prospective cohort of Chinese women. J. Nutr..

[87] Akesson A., Bjellerup P., Berglund M., Bremme K., Vahter M. (2002). Soluble transferrin receptor: Longitudinal assessment from pregnancy to postlactation. Obstetr. Gynecol..

[88] Lorenz L., Peter A., Poets C.F., Franz A.R. (2013). A review of cord blood concentrations of iron status parameters to define reference ranges for preterm infants. Neonatology.

[89] Contreras H., Chim N., Credali A., Goulding C.W. (2014). Heme uptake in bacterial pathogens. Curr. Opin. Chem. Biol..

[90] Raiten D.J., Ashour F.A.S., Ross A.C., Meydani S.N., Dawson H.D., Stephensen C.B., Brabin B.J., Ommen B.V., Suchdev P.S., Group I.C. (2014). Inflammation and nutritional science for programs/policies and interpretation of research evidence (INSPIRE). J. Nutr..

[91] Grant F.K., Suchdev P.S., Flores-Ayala R., Cole C.R., Ramakrishnan U., Ruth L.J., Martorell R. (2012). Correcting for inflammation changes estimates of iron deficiency among rural Kenyan preschool children. J. Nutr..

[92] Kaestel P., Martinussen T., Aaby P., Michaelsen K.F., Friis H. (2012). Serum retinol is associated with stage of pregnancy and the acute phase response in pregnant women in Guinea-Bissau. J. Nutr..

[93] Kuvibidila S., Vuvu M. (2009). Unusual low plasma levels of zinc in non-pregnant Congolese women. Br. J. Nutr..

[94] WHO (2011). Guideline: Intermittent Iron and Folic Acid Supplementation in Menstruating Women.

[95] WHO (2012). Guideline: Daily Iron and Folic Acid Supplementation in Pregnant Women.

[96] Nguyen P., Nava-Ocampo A., Levy A., O’Connor D.L., Einarson T.R., Taddio A., Koren G. (2008). Effect of iron content on the tolerability of prenatal multivitamins in pregnancy. BMC Pregnancy Childbirth.

[97] Pena-Rosas J.P., De-Regil L.M., Dowswell T., Viteri F.E. (2012). Daily oral iron supplementation during pregnancy. Cochrane Database Syst. Rev..

[98] Bhutta Z.A., Imdad A., Ramakrishnan U., Martorell R. (2012). Is it time to replace iron folate supplements in pregnancy with multiple micronutrients?. Paediatr. Perinatal Epidemiol..

[99] Bhutta Z.A., Ahmed T., Black R.E., Cousens S., Dewey K., Giugliani E., Haider B.A., Kirkwood B., Morris S.S., Sachdev H.P. (2008). What works? Interventions for maternal and child undernutrition and survival. Lancet.

[100] Suchdev P.S., De-Regil L.M., Walleser S., Vist G., Peña-Rosas J. (2011). Multiple Micronutrient Powders for Home (Point of Use) Fortification of Foods in Pregnant Women: A Systematic Review.

[101] Suchdev P.S., Peña-Rosas J., De-Regil L. (2014). Multiple micronutrient powders for home (point-of-use) fortification of foods in pregnant women (protocol). Cochrane Database Syst. Rev..

[102] Milman N. (2012). Postpartum anemia ii: Prevention and treatment. Ann. Hematol..

[103] De-Regil L.M., Suchdev P.S., Vist G.E., Walleser S., Pena-Rosas J.P. (2011). Home fortification of foods with multiple micronutrient powders for health and nutrition in children under two years of age. Cochrane Database Syst. Rev..

[104] Zlotkin S.H., Schauer C., Christofides A., Sharieff W., Tondeur M.C., Hyder S.M. (2005). Micronutrient sprinkles to control childhood anaemia. PLoS Med..

[105] Sazawal S., Black R.E., Ramsan M., Chwaya H.M., Stoltzfus R.J., Dutta A., Dhingra U., Kabole I., Deb S., Othman M.K. (2006). Effects of routine prophylactic supplementation with iron and folic acid on admission to hospital and mortality in preschool children in a high malaria transmission setting: Community-based, randomised, placebo-controlled trial. Lancet.

[106] Ojukwu J.U., Okebe J.U., Yahav D., Paul M. (2009). Oral iron supplementation for preventing or treating anaemia among children in malaria-endemic areas. Cochrane Database Syst. Rev..

[107] Suchdev P.S., Leeds I.L., McFarland D.A., Flores R. (2010). Is it time to change guidelines for iron supplementation in malarial areas?. J. Nutr..

[108] Iannotti L.L., Tielsch J.M., Btlack M.M., Black R.E. (2006). Iron supplementation in early childhood: Health benefits and risks. Am. J. Clin. Nutr..

[109] McDonald S.J., Middleton P., Dowswell T., Morris P.S. (2013). Effect of timing of umbilical cord clamping of term infants on maternal and neonatal outcomes. Cochrane Database Syst. Rev..

[110] Beard J. (2003). Iron deficiency alters brain development and functioning. J. Nutr..

[111] Ruel M.T., Alderman H. (2013). Nutrition-sensitive interventions and programmes: How can they help to accelerate progress in improving maternal and child nutrition?. Lancet.

